# Switching between Enzyme Replacement Therapies and Substrate Reduction Therapies in Patients with Gaucher Disease: Data from the Gaucher Outcome Survey (GOS)

**DOI:** 10.3390/jcm11175158

**Published:** 2022-08-31

**Authors:** Derralynn A. Hughes, Patrick Deegan, Pilar Giraldo, Özlem Göker-Alpan, Heather Lau, Elena Lukina, Shoshana Revel-Vilk, Maurizio Scarpa, Jaco Botha, Noga Gadir, Ari Zimran

**Affiliations:** 1 LSD Unit, Royal Free London NHS Foundation Trust, University College London, London NW3 2QG, UK; 2 Addenbrookes Hospital, Cambridge CB2 0QQ, UK; 3 CIBER de Enfermedades Raras, IIS Aragon, 50009 Zaragoza, Spain; 4 Translational Research Unit, IIS Aragon, 50009 Zaragoza, Spain; 5 Lysosomal Disorders Unit and Center for Clinical Trials, O&O Alpan LLC, Fairfax, VA 22030, USA; 6 Department of Neurology, New York University School of Medicine, New York, NY 10016, USA; 7 Department of Orphan Diseases, National Research Center for Hematology, 125167 Moscow, Russia; 8 Gaucher Unit, Shaare Zedek Medical Center, Jerusalem 9103102, Israel; 9 The Faculty of Medicine, Hebrew University, Jerusalem 9112102, Israel; 10 Centre for Rare Diseases, Academic Medical Centre Hospital of Udine, 33100 Udine, Italy; 11 Takeda Pharmaceuticals International AG, 8152 Zurich, Switzerland

**Keywords:** Gaucher disease, enzyme replacement therapy, substrate reduction therapy, treatment switch

## Abstract

Switching between enzyme replacement therapies (ERT) and substrate reduction therapies (SRT) in patients with type 1 Gaucher disease (GD1) is not uncommon; however, the reasons for switchng treatments have not been explored in detail. Data from the Gaucher Outcome Survey (GOS), an international registry for patients with confirmed GD, were used to evaluate the reasons for, and consequences of, switching between these treatment types. Of the 1843 patients enrolled in GOS on 25 February 2020, 245 had undergone a treatment switch: 222 from initial ERT to SRT (of whom 88 later switched back to ERT) and 23 from initial SRT to ERT. The most common reasons for ERT–SRT switching were duration of infusion (25.4%), drug shortage (22.0%), and adverse events (AEs; 11.9%), and for SRT–ERT switching, AEs (63.6%), lack of beneficial effect (16.4%), and participation in a clinical trial (9.1%). Bodyweight and hematologic parameters largely remained stable before and after switching between ERT and SRT, although with substantial variation between patients. These findings contribute to understanding why treatment switching occurs in patients with GD, and may help physicians recognize the real-world impact of treatment switching between ERT and SRT for patients with GD.

## 1. Introduction

Gaucher disease (GD) is a rare, inherited metabolic disorder caused by mutations in the gene for the lysosomal enzyme β-glucocerebrosidase. Deficiency of this enzyme activity results in the accumulation of glycosphingolipids in lysosomes—predominantly glucocerebroside—in cells of the monocyte–macrophage system and leads to multisystemic disease manifestations, including splenomegaly, hepatomegaly, thrombocytopenia, anemia, osteopenia/osteoporosis, osteonecrosis, and bone pain [1,2]. Although there is some phenotypic overlap, particularly between neuronopathic forms, patients are typically categorized into three disease types (1, 2 and 3) on the basis of the presence and severity of specific neurological manifestations [1,2].

Enzyme replacement therapy (ERT) and substrate reduction therapy (SRT) can both reduce the accumulation of glucocerebrosidase, either by augmenting the defective β-glucocerebrosidase enzyme (in the case of ERT) or by partial inhibition of glucocerebroside synthesis from ceramide (in the case of SRT) [1]. Although direct clinical comparisons between agents have not been made between ERT and SRT, both classes have been shown to result in improvements in systemic, but not neurologic [3,4], manifestations of GD [5,6,7,8]. Six agents from these classes have been approved for the treatment of Gaucher disease type 1 (GD1). The ERTs alglucerase and imiglucerase, first approved in the 1990s, were followed by velaglucerase alfa in 2010 and taliglucerase alfa in 2012, the latter not available in the EU at the time of publication. SRT is not currently approved for use in pediatric patients (<18 years of age in the US and <16 years in the UK). Miglustat, the first SRT for GD1, was approved in the early 2000s for adult patients as a second-line therapy after ERT has been deemed unsuitable [9]. This was followed by eliglustat, first approved in 2014 for adults with GD1 who are CYP2D6 extensive metabolizers, intermediate metabolizers, or poor metabolizers, as determined by an FDA-approved test, including for use as first-line therapy [10].

For patients with long-term conditions, the decision to switch to a different treatment may be influenced by multiple factors, including inadequate efficacy, the presence of adverse events, adherence to treatment, or reimbursement reasons associated with high treatment costs [11,12], with estimated annual drug costs ranging from EUR 124,000 to EUR 258,000 per patient (2011 data) [13]. A survey of attitudes toward treatments amongst patients with GD revealed significant variation in the perception of ERT and SRT, with side-effects reported as the most common concern regarding SRTs and the inconvenience of regular infusions as a common concern related to ERTs [14]. With the approval of each additional agent, the opportunity to switch treatments becomes available to more patients. Few studies, however, have explored in detail the reasons for switching between ERTs and SRTs.

To better understand the factors influencing switching from an ERT to an SRT, or vice versa, an analysis of data from the Gaucher Outcome Survey (GOS) patient registry was conducted to investigate the characteristics of patients who switched treatments, the reasons for switching between treatment classes, and the consequences of switching treatments among individuals enrolled in GOS.

## 2. Materials and Methods

### 2.1. Study Design

GOS (NCT03291223) is an ongoing disease-specific registry initiated in September 2010 by Shire, a Takeda company, for patients with a confirmed biochemical or genetic diagnosis of GD, regardless of treatment status or type of treatment received, and who are not actively participating in a clinical trial for GD. While enrolled in GOS, patients receive treatment as per investigator judgement and local guidelines and standards. The objectives of the registry include evaluation of the safety and long-term effectiveness of velaglucerase alfa, and characterization of patients receiving velaglucerase alfa or other GD-specific treatments, to gain a better understanding of the natural history of GD and to serve as a database for evidence-based management of GD over time in real-world clinical practice. Written informed consent is required for participation in GOS. For patients <18 years of age (<16 years of age in the UK), consent is obtained from a parent or legal representative along with assent, where appropriate.

This retrospective patient registry analysis of patterns of GD-specific ERT and SRT treatment switching was conducted using data for patients with GD of any type who had enrolled in GOS, who received treatment with any ERT or any SRT and who had recorded at least one switch between an ERT and an SRT or vice versa, before 25 Feb 2020.

### 2.2. Data Collection

Patient data from routine visits were collected via web-based electronic case report forms (eCRF); in addition, data preceding GOS enrollment were used retrospectively when available. Data were handled in accordance with relevant global and local regulations and best practice and with Good Pharmaco-epidemiological Practice (GPP), Good Research for Comparative Effectiveness principles, and the principles of the International Conference on Harmonization Good Clinical Practice (GCP) guidelines.

Information on patient demographics, diagnosis, treatment types and doses, dates of switching, change over time in hemoglobin concentration, platelet count, and body weight, and the number and type of AEs was extracted from GOS for this analysis. AEs were recorded against the treatment type at the time of onset, regardless of switching status or time since switching from the previous treatment (AEs were defined as any undesirable experience occurring whilst in receipt of ERT or SRT).

### 2.3. Data Analyses

Baseline was defined as the start of any GD-specific treatment, either before or after enrollment into GOS. Reasons for switching were analyzed by category as entered by each participating center; reasons given as “other” were categorized manually based on free-text information, if available. Data for continuous variables were presented using descriptive statistics. For categorical variables, the number and percentage of patients in each category (including a missing category, if applicable) were reported. Percentages were calculated using the number of patients with available data as the denominator. Statistical analyses were performed using SAS^®^ software version 9.4 (SAS Institute, Cary, NC, USA).

## 3. Results

### 3.1. Patient Demographics

**Figure 1:**
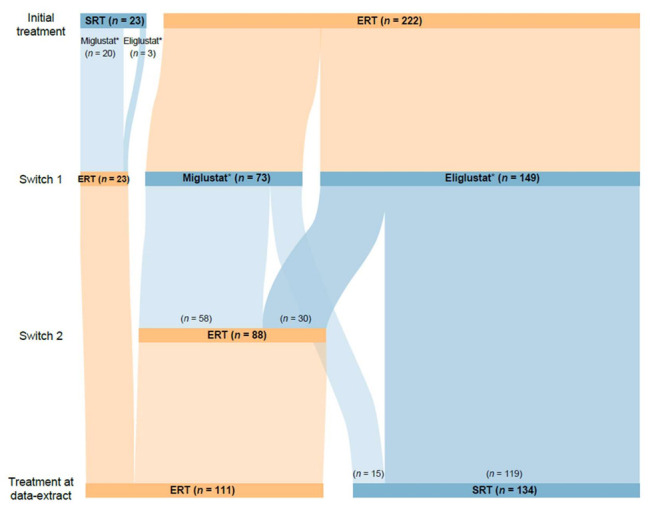
Patient flow. Abbreviations: ERT, enzyme replacement therapy; SRT, substrate reduction therapy. * Treatments indicate the first agent received in each treatment period. Subsequent within-class switches were not recorded.

Of 1843 patients enrolled in GOS at the time of this analysis, 1388 had received GD-specific treatment. A total of 1120 of these patients had received at least one agent of a single treatment type (1076 received ≥1 ERT only (689 imiglucerase, 606 velaglucerase alfa, 114 taliglucerase alfa, 108 alglucerase), 42 received ≥1 SRT only (33 eliglustat, 10 miglustat), and two “other” treatments) and 245 had switched between ERT and SRT treatments. Of those who had switched, 23 (13 male and 10 female) initiated treatment with an SRT (20 miglustat, 3 eliglustat) and switched to an ERT, and 222 (96 male and 126 female) initiated treatment with an ERT (143 imiglucerase, 48 alglucerase, 27 velaglucerase alfa, 4 taliglucerase alfa) and switched to an SRT. Within the ERT–SRT group, 88 patients (35 male and 53 female) later switched back to ERT, while 134 (61 male and 73 female) remained on SRT to the end of the analysis period (Figure 1). A further 23 patients who underwent alternative switching patterns were excluded from this analysis owing to small patient numbers in each cohort.

Most of the 245 patients included in this analysis (98.3% (*n* = 236 of 240 with available data)) were diagnosed with GD1 (Table 1). Patients first treated with ERT had a younger mean age at treatment start than those who initiated treatment with SRT (31.2 vs. 42.9 years, respectively), were younger at diagnosis (17.9 vs. 30.2 years) and had a shorter delay in diagnosis (1.7 vs. 5.5 years). A total of 59 (24.1%) patients started treatment at age <18 years, all of whom had ERT as their first treatment. All 23 patients starting with SRT initiated treatment at age ≥18 years.

### 3.2. Patterns of Treatment Switching

**Figure 2:**
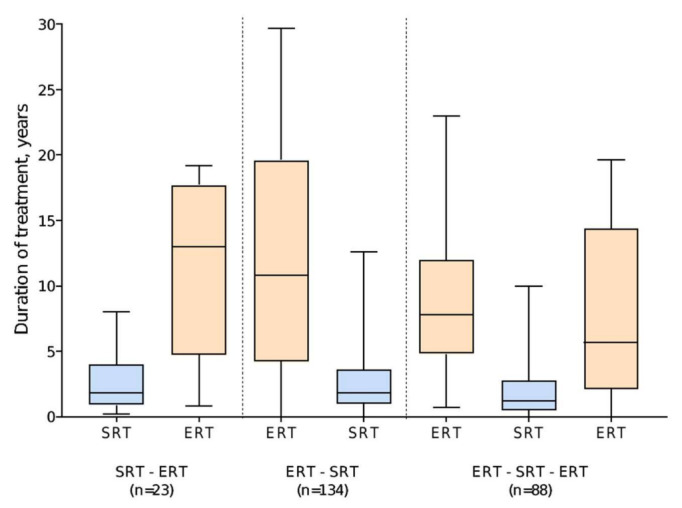
Duration of therapy by treatment type.

Of 222 patients who started treatment with ERT, 149 switched to eliglustat and 73 switched to miglustat. The median duration of ERT was 14.1 years (range 0 (single dose) to 29.7 years), compared with 1.6 years (range 0 (single dose) to 12.6 years) for SRT after switching. Of the 88 patients who subsequently switched back to ERT, 30 (34.1%) switched back from eliglustat and 58 (65.9%) from miglustat. This group tended to have shorter durations of SRT (median 1.2 years (range 0 (single dose) to 10.0 years), *n* = 88) than those who remained on SRT to the end of the analysis period (median 1.8 years [range 0–12.6 years], *n* = 134) (Figure 2).

Of 23 patients who started treatment with an SRT, 20 were receiving miglustat at the time of switching to ERT and three were receiving eliglustat. The median duration of initial SRT was 1.8 years (range 0.2–8.0), with the subsequent switch to ERT occurring prior to the approval of eliglustat (2014 (US) or 2015 (UK, Israel)) for most patients. The median duration of ERT as second therapy type in this group of patients was 13.0 years (range 0.8–19.2) (Figure 2).

In total, 134 patients were receiving SRT at the end of the analysis period (Figure 1); of these, 15 had switched from ERT to miglustat; seven later switched to eliglustat and eight were still receiving miglustat at the end of the analysis. All eight patients remaining on miglustat (treatment duration 7.9 [2.2–20.0] years) were diagnosed with GD1, six were female, and all were widely geographically distributed, residing in Canada, the UK, Israel, and Spain.

### 3.3. Reasons for Switching

**Figure 3:**
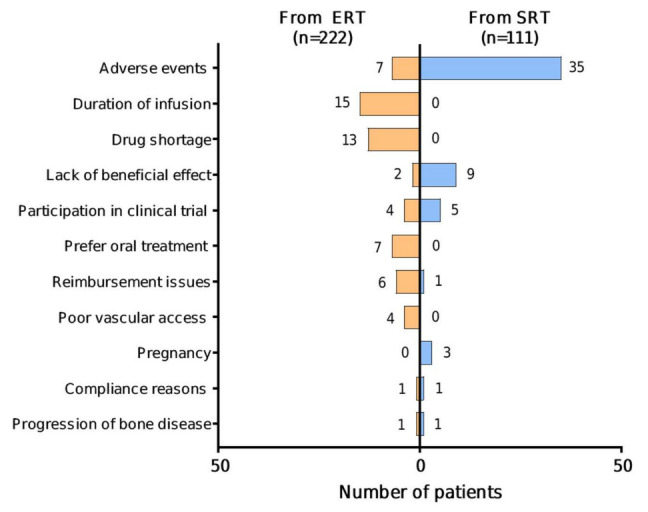
Reasons for switching.

Specific reasons for switching were provided for 59/222 (26.6%) patients for ERT—SRT switch (total 60 reasons; two reasons provided for one patient), and for 55/111 (49.5%) patients for SRT—ERT switch. Where provided, specific reasons given for switching from ERT to SRT were: duration of infusion (15/59 (25.4%)); the imiglucerase drug shortage in 2009 (13/59 (22.0%)); AEs (7/59 (11.9%)); preference for oral treatment (7/59 (11.9%)); reimbursement issues (6/59 (10.2%)); poor vascular access (4/59 (6.8%)); participation in a clinical trial (4/59 (6.8%)); lack of beneficial effect (2/59 (3.4%)); compliance reasons (1/59 (1.7%)); and progression of bone disease (1/59 (1.7%)) (Figure 3). Specific AEs given as a reason for switching from ERT to SRT were provided for 1/7 patients only (fracture). Reasons for switching from ERT were similar irrespective of which SRT patients switched to, with notable exceptions of the imiglucerase drug shortage (11 patients to miglustat vs. two to eliglustat) and clinical trial participation (none to miglustat vs. 47 to eliglustat), both related to the time-period in which these switches took place. Switches from ERT to SRT because of lack of beneficial effect (*n* = 2) or progression of bone disease (*n* = 1) were noted to occur at below maximal ERT doses (<50 U/kg) for all three patients.

Specific reasons for switching from SRT to ERT were: AEs (35/55 (63.6%)); lack of beneficial effect (9/55 (16.4%)); participation in a clinical trial (5/55 (9.1%)); pregnancy (3/55 (5.5%)); compliance reasons; reimbursement issues; and progression of bone disease (1/55 (1.8%) for each) (Figure 3). As is standard for patient registries, pregnancy was included as an AE in the GOS registry for the purpose of data collection. Specific AEs given as a reason for switching from SRT to ERT were provided for 29/35 patients and included gastrointestinal symptoms (*n* = 5), skin related AEs (*n* = 5), dizziness (*n* = 3), drug intolerance (*n* = 3), fatigue (*n* = 2), change in taste (*n* = 2), and one case each of chills, cough, disorientation, general malaise, fracture, headache, poor balance, purpuric dermatosis, and moderate thrombocytopenia.

### 3.4. Impact of Drug Availability

The availability of a second SRT option appeared to influence the decision to switch treatment. Information relating to the timing of switching was available for 208/222 patients who switched from ERT to SRT and for 109/111 patients who switched from SRT to ERT. A total of 62/83 (74.7%) patients who switched from ERT before the first eliglustat approval (August 2014) switched to miglustat, compared with 117/125 (93.6%) patients who switched from ERT to eliglustat after this date. Prior to February 2010, the date of first approval of velaglucerase alfa, 44/58 (75.9%) switches from SRT to ERT were from miglustat to imiglucerase, compared with 10/58 (17.2%) from miglustat to velaglucerase alfa, 3/58 (5.2%) from miglustat to taliglucerase alfa and 1/58 (1.7%) from eliglustat to imiglucerase. After May 2012, when velaglucerase alfa, taliglucerase alfa, and eliglustat were all approved, 29/41 (70.7%) switches from SRT to ERT were from eliglustat to ERT (19 imiglucerase, nine velaglucerase alfa, and one taliglucerase alfa), and 12/41 (29.3%) were from miglustat to ERT (six imiglucerase, four velaglucerase alfa, and two taliglucerase alfa).

### 3.5. Adverse Events

Overall, 226 AEs were reported by patients included in this analysis over the follow-up period: 139 in 51 patients over 13.6 years of ERT, and 87 in 36 patients over 2.4 years of SRT (Table 2). In total, eight AEs considered related to treatment occurred in three patients (1.2%) while on ERT, and 48 treatment-related events occurred in 21 patients (8.6%) while on an SRT, most of which were associated with eliglustat (46 events in 20 patients). AEs were given as the reason for switching for 23.1% of patients (18/78) treated with miglustat and 51.5% of patients (17/33) treated with eliglustat. AEs leading to a switch from eliglustat were most commonly related to gastrointestinal symptoms (9 events), skin and subcutaneous tissue disorders (8 events), or nervous system disorders (6 events). Information about specific AEs resulting in treatment switching were not captured in the database for patients switching from miglustat to ERT. AEs were more likely to result in a switch from SRT to ERT in patients who had previously switched from ERT to SRT than in those for whom SRT was their initial therapy (32 (36.4%) vs. 3 (13.0%) patients).

Overall, seven patients died during the evaluation period: four while receiving ERT (two imiglucerase and two velaglucerase alfa), and three while receiving SRT (all miglustat). Reasons for death were given as stroke, sepsis, and respiratory failure (imiglucerase, *n* = 2); pancreatic cancer and pulmonary edema (velaglucerase alfa, *n* = 2); and multiple myeloma and two cases of respiratory failure (miglustat, *n* = 3), none of which were considered related to treatment.

### 3.6. Hematologic Outcomes

**Figure 4:**
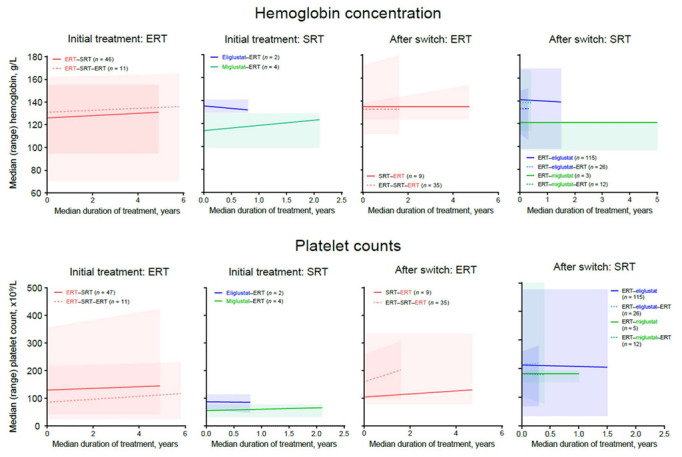
Change in median (range) hemoglobin concentrations and platelet counts before and after switch.

Hemoglobin concentrations increased or remained stable in patients with available data who were receiving ERT or miglustat at any time. A small, clinically insignificant decline in hemoglobin concentration was observed in four patients receiving eliglustat as an initial therapy, although all patients remained above the lower value of the normal range (Figure 4). Improvement or stability in mean platelet counts was observed with ERT and SRT in all treatment periods (Figure 4). Within the SRT–ERT group, lower baseline hemoglobin concentrations and platelet counts were observed for patients switching from miglustat (median [range] 114.0 [99.0–129.0] g/L and 56.0 [34.0–78.0] × 10^9^/L, *n* = 4) than in those switching from eliglustat (135.5 [130.0–141.0] g/L and 88.0 [61.0–115.0] × 10^9^/L, *n* = 2) (Figure 4).

### 3.7. Change in Bodyweight

Data for change in bodyweight were available for 39 patients during treatment with ERT and for 79 patients during treatment with SRT (72 patients with eliglustat, seven patients with miglustat). Overall, mean bodyweight remained stable over time with all treatments. Mean change in bodyweight was 3.7 kg over 3.8 years with ERT (3.4 kg/4.7 years with ERT as first treatment, and 3.9 kg/2.8 years with ERT post-switch), and 0.4 kg/1.6 years with SRT (0.2 kg/1.9 years with SRT as first treatment and 0.4 kg/1.6 years with SRT post-switch).

There was considerable variation in bodyweight change within treatment groups. With ERT, bodyweight changes ranged from −12.9 to +23.8 kg over treatment durations ranging from 0.9 to 23.8 years. With SRT, bodyweight changes ranged from −40.1 kg (in an individual with a baseline weight of 130.6 kg) over 3.7 years to +20 kg (in an individual with a baseline weight of 76.2 kg) over 5 years, both with eliglustat. Less extreme changes were observed with miglustat, with bodyweight changes ranging from −19.5 kg to +2.2 kg over treatment durations ranging from 0.3 to 4.0 years.

## 4. Discussion

ERT has long been the mainstay of therapy for patients with GD, but as treatment options expand with the development of additional GD-specific agents, the opportunity to switch between treatment modalities (i.e., ERT infusions vs. oral SRT) has increased. However, although the clinical efficacy of individual agents after switching has been explored in clinical trials and studies of real-world data [15,16,17,18,19,20], the reasons for switching between ERT and SRT in patients with GD have not previously been evaluated in detail. Although clinical trials generate valuable evidence for the efficacy and safety of individual treatments before and after switching, the drivers leading to treatment switching cannot be explored in a clinical trial setting. Disease registries, despite their intrinsic limitations, provide a valuable source of real-world data that can be interrogated to assess real-world treatment practices. This analysis using data from GOS was conducted to understand the reasons underlying switching from one therapeutic approach to another (ERT to SRT or vice versa) in patients with GD. Notwithstanding an inherent bias toward the inclusion of patients receiving ERT in a registry sponsored by an ERT manufacturer, and the longer availability of ERT, GOS provides data to enable the exploration of real-world treatment patterns among an international sample of patients with GD. This analysis included data up to 25 February 2020, prior to the SARS-CoV-2 pandemic.

Our findings revealed that reasons for switching from ERT to SRT were approximately equally divided between factors relating to the treatment itself (i.e., duration of infusion, poor vascular access, AEs, or lack of beneficial effect), accounting for 28 of 60 switches (46.7%), and additional factors (i.e., the imiglucerase drug shortage in 2009, reimbursement issues, or a preference for oral treatment), accounting for 26 of 60 switches (43.3%). Conversely, reasons given for switching from SRT to ERT were predominantly related to the treatment itself: AEs, lack of beneficial effect, pregnancy, progression of bone disease, and compliance reasons accounted for 49 of 55 switches (89.1%), while participation in a clinical trial and reimbursement issues accounted for six of 55 switches (10.9%). The reasons for switching from ERT to SRT were similar for both miglustat and eliglustat, excepting those related to the date of switching (imiglucerase shortage and eliglustat clinical trial recruitment), suggesting that the driver for switching lies with a desire for an alternative route of administration (intravenous to oral), and may be influenced by the treating physician’s preference or may be related to availability of the agent.

AEs were more frequently cited as the reason for switching from SRT than from ERT (63.6% vs. 11.9% switches), although the overall occurrence of any AEs over the respective mean treatment periods was higher with ERT (20.8% of patients over 13.6 years) than with SRT (14.7% of patients over 2.4 years). Certain types of AE, notably infusion reactions, diarrhea, nausea and headache, are known to occur shortly after treatment initiation [21,22], preventing comparison of annual rates between treatment cohorts in this analysis. Different treatment modalities are associated with different types of AEs, as indicated in the respective product labels. The most common AEs associated with miglustat are diarrhea, flatulence, abdominal pain, weight loss, and tremor [9], whereas gastrointestinal effects are also common with eliglustat [10]. In contrast, hypersensitivity is the most common AE associated with infusion-administered ERT [23,24,25]. As SRTs do not include an active biologic, they are less likely to trigger an immune reaction.

A higher proportion of patients in the GOS registry switched from an initial ERT to an SRT than from an initial SRT to an ERT. However, this should be considered in the context of the data source, whereby a potential selection bias for the inclusion of patients receiving ERT as starting therapy may exist, combined with the fact that ERT was the first disease-modifying therapy available to patients with GD, with the choice to switch to an oral agent becoming available later. Further, SRT is restricted to adult patients [9,10], necessitating the use of ERT as initial therapy for patients <18 years of age. Specific reasons for switching were not provided for a large proportion of switches, particularly from miglustat to ERT, highlighting the need to interpret these findings with care.

The availability of ERT and SRT may have played a role in initial treatment selection and subsequent decisions to switch. Although both ERT and SRT are now well established, the first two ERT agents were approved around a decade prior to the first SRT, precluding between-class switching outside clinical trial participation during this time. Additionally, the more restrictive population for whom miglustat is indicated further reduced the pool of patients potentially available switch to SRT until the later approval of eliglustat. Although patients actively participating in clinical trials for GD-specific drugs are excluded from GOS, retrospective data from prior trial participation were not excluded from this analysis. Prior to the approval of eliglustat in 2014, a large proportion of patients who switched to an SRT (mostly miglustat) later switched back to ERT, whereas patients switching to an SRT after this date were more likely to switch to eliglustat and to remain on this agent up to the end of the analysis period in February 2020. In contrast, few patients remained on miglustat to the end of the follow-up period. Further, the imiglucerase drug shortage in 2009 may have driven additional switches to alternative treatments where a switch might otherwise not have been considered [26].

Maintenance or improvement in hematologic outcomes are a key measure for GD-specific treatment and were evaluated before and after switching. In this study, improvement or stabilization in mean hemoglobin concentrations and platelet counts were observed for all treatment periods with ERT and most with SRT; however, there was a broad range of responses, with some patients experiencing declines, both before and after switching treatment, and others seeing marked improvements. It is important to note that these findings reflect outcomes for subsets of patients with unsatisfactory responses to their treatment and does not indicate general experience with these agents.

Changes in bodyweight can be a concern for some patients using ERT and SRT [27]. In this analysis, bodyweight remained stable for most patients. For those whose weight increased, larger increases were observed with ERT than with SRT. Consistent with the AE profile for miglustat, most patients receiving miglustat reported weight loss or minimal weight gain, in contrast to wide variation in weight change observed for patients receiving eliglustat. However, as observed with hemoglobin concentrations and platelet counts, there was substantial variation between individual patients in each treatment group.

Limitations of the study include those inherently associated with registry analyses. Because data are collected during routine clinical practice, the frequency of visits and the assessments carried out at each visit can vary considerably between patients. Further, participation in GOS is voluntary and populations may be susceptible to participation bias. Unlike other registries, GOS includes a data validation step designed to maximize the quality of collected data; nonetheless, the resulting data may be incomplete and subject to data entry errors. Retrospective data relating to any GD-specific treatments received prior to inclusion in GOS may not be available for all patients, which may be pertinent for those for whom initial treatment was indicated as being miglustat. This analysis did not evaluate switching that may have occurred within treatment classes (i.e., between alglucerase, imiglucerase, velaglucerase alfa, and taliglucerase alfa and between miglustat and eliglustat). Further analysis could help determine whether patients typically switched to another ERT prior to switch to SRT. Additionally, the impact of treatment switching on amelioration of hepatosplenomegaly, an important treatment goal for GD, was not explored here.

## 5. Conclusions

Although trends were apparent in reasons for switching from ERT or SRT, these reasons were predominantly related to individual experiences, and broad variation in those safety and efficacy outcomes assessed in this study were observed after switching. Shared decision making to determine an individualized approach to treatment choices and subsequent decisions to switch treatment should take into consideration multiple factors, including the efficacy and safety profiles of individual treatments and the patient’s age, family planning, personal circumstances, tolerance to potential side-effects, drug availability, and cost/reimbursement.

## Figures and Tables

**Table 1:** Patient demographics at baseline.

	ERT–SRT	SRT–ERT
	(*n* = 222)	(*n* = 23)
Ethnicity, *n* (%)		
*n* (missing)	180 (42)	21 (2)
Ashkenazi Jewish	85 (47.2)	19 (90.5)
Other	95 (52.8)	2 (9.5)
Age at diagnosis, years		
*n* (missing)	199 (23)	23
Mean (SD)	17.9 (13.9)	30.2 (17.52)
Median (range)	15.5 (0.0–63.9)	27.7 (4.0–56.5)
Sex, *n* (%)		
*n* (missing)	222	23
Male	96 (43.2)	13 (56.5)
Female	126 (56.8)	10 (43.5)
Age at ERT/SRT start, years		
*n* (missing)	222	23
Mean (SD)	31.2 (16.2)	42.9 (12.58)
Median (range)	31.3 (0.7–76.2)	47.3 (22.8–62.8)
Delay in diagnosis, years *		
*n* (missing)	160 (62)	21 (2)
Mean (SD)	1.7 (5.53)	5.5 (13.5)
Median (range)	0 (−14.1, 37.0)	0 (−13.0, 45.6)
Time on ERT, years ^†^		
*n* (missing)	222	23
Mean (SD)	13.8 (8.1)	11.1 (6.71)
Median (range)	14.1 (0.0 ^‡^–29.7)	13.0 (0.8–19.2)
Time on SRT, years ^†^		
*n* (missing)	222	23
Mean (SD)	2.4 (2.4)	2.5 (1.96)
Median (range)	1.6 (0.0 ^‡^–12.6)	1.8 (0.2–8.0)
Gaucher disease type, *n* (%)		
*n* (missing)	217 (5)	23
1	213 (98.2)	23 (100)
2	1 (0.5)	0
3	3 (1.4)	0
Mutation, *n* (%)		
*n* (missing)	143 (79)	22 (1)
N370S/N370S	54 (37.8)	12 (54.5)
N370S/Other	73 (51.0)	7 (31.8)
L444P/L444P	4 (2.8)	1 (4.5)
L444P/Other	9 (6.3)	0
Other	3 (2.1)	2 (9.1)
Country, *n* (%)		
*n* (missing)	222	23
US	95 (42.8)	4 (17.4)
Israel	53 (23.9)	15 (65.2)
UK	42 (18.9)	2 (8.7)
Other	32 (14.4)	2 (8.7)
Splenectomy status, *n* (%)		
*n* (missing)	222	23
Splenectomized	48 (21.6)	4 (17.4)
Non-splenectomized	174 (78.4)	19 (82.6)

Abbreviations: SD, standard deviation. * Time from symptom onset to diagnosis; includes patients diagnosed prior to symptom onset, e.g., as a result of family history or genetic testing. ^†^ Duration of any ERT/SRT at any time, irrespective of switch order. ^‡^ Patient received a single dose prior to switch.

**Table 2:** Summary of patients experiencing adverse events.

Patients with:	ERT (*n* = 245)	SRT (*n* = 245)	
		Eliglustat (*n* = 152)	Miglustat (*n* = 93)
Any AE, *n* (%)	51 (20.8)	28 (18.4)	8 (8.6)
AE related to treatment, *n* (%)	3 (1.2)	20 (13.2)	1 (1.1)
Serious AE, *n* (%)	31 (12.7)	7 (4.6)	3 (3.2)
Serious AE related to treatment, *n* (%)	0	2 (1.3) *	0
Fatal AEs, *n* (%)	4 (1.6)	0	3 (3.2)

Abbreviation: AE, adverse event. * Acute myocardial infarction and pregnancy (pregnancies are recorded in the GOS database, pregnancy complications such as a spontaneous abortion/miscarriage or congenital abnormality were considered SAEs).

## Data Availability

The datasets, including the redacted study protocol, redacted statistical analysis plan, and individual participant data supporting the results reported in this article, will be made available within three months from initial request to researchers who provide a methodologically sound proposal. The data will be provided after de-identification, in compliance with applicable privacy laws, data protection, and requirements for consent and anonymization.

## Supplementary Materials

The following supporting information can be downloaded at: www.mdpi.com/article/10.3390/jcm11175158/s1, Table S1. GOS Steering Committee members; Table S2. GOS IRB locations.

## References

[1] Revel-Vilk S., Szer J., Zimran A., Kaushansky K., Lichtman M., Prchal J., Levi M., Burns L.J., Linch D. (2021). Gaucher disease and related lysosomal storage diseases. Williams Hematology.

[2] Goker-Alpan O., Schiffmann R., Park J.K., Stubblefield B.K., Tayebi N., Sidransky E. (2003). Phenotypic continuum in neuronopathic Gaucher disease: An intermediate phenotype between type 2 and type 3. J. Pediatr..

[3] Bennett L.L., Fellner C. (2018). Pharmacotherapy of Gaucher disease: Current and future options. Pharm. Ther..

[4] Li M. (2018). Enzyme replacement therapy: A review and its role in treating lysosomal storage diseases. Pediatr. Ann..

[5] Zimran A., Durán G., Giraldo P., Rosenbaum H., Giona F., Petakov M., Terreros Muñoz E., Solorio-Meza S.E., Cooper P.A., Varughese S. (2019). Long-term efficacy and safety results of taliglucerase alfa through 5years in adult treatment-naïve patients with Gaucher disease. Blood Cells Mol. Dis..

[6] Kishnani P.S., DiRocco M., Kaplan P., Mehta A., Pastores G.M., Smith S.E., Puga A.C., Lemay R.M., Weinreb N.J. (2009). A randomized trial comparing the efficacy and safety of imiglucerase (Cerezyme) infusions every 4 weeks versus every 2 weeks in the maintenance therapy of adult patients with Gaucher disease type 1. Mol. Genet. Metab..

[7] Hughes D.A., Gonzalez D.E., Lukina E.A., Mehta A., Kabra M., Elstein D., Kisinovsky I., Giraldo P., Bavdekar A., Hangartner T.N. (2015). Velaglucerase alfa (VPRIV) enzyme replacement therapy in patients with Gaucher disease: Long-term data from phase III clinical trials. Am. J. Hematol..

[8] Mistry P.K., Lukina E., Ben Turkia H., Shankar S.P., Baris H., Ghosn M., Mehta A., Packman S., Pastores G., Petakov M. (2017). Outcomes after 18 months of eliglustat therapy in treatment-naïve adults with Gaucher disease type 1: The phase 3 ENGAGE trial. Am. J. Hematol..

[9] Actelion Pharmaceuticals ZAVESCA® (Miglustat) Capsules, for Oral Use. Prescribing Information. https://www.accessdata.fda.gov/drugsatfda_docs/label/2014/021348s010lbl.pdf.

[10] Genzyme CERDELGA™ (Eliglustat) Capsules, for Oral Use. Prescribing Information. https://www.accessdata.fda.gov/drugsatfda_docs/label/2018/205494Orig1s000lbl.pdf.

[11] Mäurer M., Tiel-Wilck K., Oehm E., Richter N., Springer M., Oschmann P., Manzel A., Hieke-Schulz S., Zingler V., Kandenwein J.A. (2019). Reasons to switch: A noninterventional study evaluating immunotherapy switches in a large German multicentre cohort of patients with relapsing-remitting multiple sclerosis. Ther. Adv. Neurol. Disord..

[12] Luttropp K., Dalén J., Svedbom A., Dozier M., Black C.M., Puenpatom A. (2020). Real-world patient experience of switching biologic treatment in inflammatory arthritis and ulcerative colitis—a systematic literature review. Patient Prefer. Adherence.

[13] Van Dussen L., Biegstraaten M., Hollak C.E.M., Dijkgraaf M.G.W. (2014). Cost-effectiveness of enzyme replacement therapy for type 1 Gaucher disease. Orphanet J. Rare Dis..

[14] Wagner V.F., Northrup H., Hashmi S.S., Nguyen J.M., Koenig M.K., Davis J.M. (2018). Attitudes of individuals with Gaucher disease toward substrate reduction therapies. J. Genet. Couns..

[15] Smith L., Rhead W., Charrow J., Shankar S.P., Bavdekar A., Longo N., Mardach R., Harmatz P., Hangartner T., Lee H.M. (2016). Long-term velaglucerase alfa treatment in children with Gaucher disease type 1 naïve to enzyme replacement therapy or previously treated with imiglucerase. Mol. Genet. Metab..

[16] Pleat R., Cox T.M., Burrow T.A., Giraldo P., Goker-Alpan O., Rosenbloom B.E., Croal L.R., Underhill L.H., Gaemers S.J., Peterschmitt M.J. (2016). Stability is maintained in adults with Gaucher disease type 1 switched from velaglucerase alfa to eliglustat or imiglucerase: A sub-analysis of the eliglustat ENCORE trial. Mol. Genet. Metab. Rep..

[17] Zimran A., Gonzalez-Rodriguez D.E., Abrahamov A., Cooper P.A., Varughese S., Giraldo P., Petakov M., Tan E.S., Chertkoff R. (2018). Long-term safety and efficacy of taliglucerase alfa in pediatric Gaucher disease patients who were treatment-naïve or previously treated with imiglucerase. Blood Cells Mol. Dis..

[18] Giraldo P., Andrade-Campos M., Alfonso P., Irun P., Atutxa K., Acedo A., Barez A., Blanes M., Diaz-Morant V., Fernández-Galán M.A. (2018). Twelve years of experience with miglustat in the treatment of type 1 Gaucher disease: The Spanish ZAGAL project. Blood Cells Mol. Dis..

[19] Mistry P.K., Balwani M., Charrow J., Kishnani P., Niederau C., Underhill L.H., McClain M.R. (2020). Real-world effectiveness of eliglustat in treatment-naive and switch patients enrolled in the International Collaborative Gaucher Group Gaucher Registry. Am. J. Hematol..

[20] Kleytman N., Ruan J., Ruan A., Zhang B., Murugesan V., Lin H., Guo L., Klinger K., Mistry P.K. (2021). Incremental biomarker and clinical outcomes after switch from enzyme therapy to eliglustat substrate reduction therapy in Gaucher disease. Mol. Genet. Metab. Rep..

[21] Barbey F., Livio F., Mehta A., Beck M., Sunder-Plassmann G. (2006). Safety of enzyme replacement therapy. Fabry Disease: Perspectives from 5 Years of FOS.

[22] Peterschmitt M.J., Freisens S., Underhill L.H., Foster M.C., Lewis G., Gaemers S.J.M. (2019). Long-term adverse event profile from four completed trials of oral eliglustat in adults with Gaucher disease type 1. Orphanet J. Rare Dis..

[23] Genzyme Corporation Cerezyme (Imiglucerase for Injection). Prescribing Information. https://www.accessdata.fda.gov/drugsatfda_docs/label/2005/20367s066lbl.pdf.

[24] Shire Human Genetic Therapies VPRIV® (Velaglucerase Alfa for Injection), for Intravenous Use. Prescribing Information. https://www.accessdata.fda.gov/drugsatfda_docs/label/2019/022575s022lbl.pdf.

[25] Pfizer Eleyso™ (Taliglucerase Alfa) for Injection, for Intravenous Use. Prescribing Information. https://www.accessdata.fda.gov/drugsatfda_docs/label/2014/022458s003s006lbl.pdf.

[26] Hollak C.E., vom Dahl S., Aerts J.M., Belmatoug N., Bembi B., Cohen Y., Collin-Histed T., Deegan P., van Dussen L., Giraldo P. (2010). Force majeure: Therapeutic measures in response to restricted supply of imiglucerase (Cerezyme) for patients with Gaucher disease. Blood Cells Mol. Dis..

[27] Langeveld M., de Fost M., Aerts J.M., Sauerwein H.P., Hollak C.E. (2008). Overweight, insulin resistance and type II diabetes in type I Gaucher disease patients in relation to enzyme replacement therapy. Blood Cells Mol. Dis..

