# Supplementary material for: Switching between Enzyme Replacement Therapies and Substrate Reduction Therapies in Patients with Gaucher Disease: Data from the Gaucher Outcome Survey (GOS)
Source: J Clin Med. 2022 Aug 31;11(17):5158. doi: 10.3390/jcm11175158 (PMC9457166; doi:10.3390/jcm11175158)
Supplement: Supplementary file 1 [file jcm-11-05158-s001.zip › jcm-1840988-supplementary.pdf]

Supplementary Materials

Table S1. GOS Steering Committee members

Tanya Collin-Histed
Patrick Deegan
Deborah Elstein
Diego Fernández-Sasso
Pilar Giraldo
Özlem Göker-Alpan
Derralynn Hughes
Heather Lau*
Elena Lukina
Shoshana Revel-Vilk
Maurizio Scarpa
Ida Schwartz
Stephan vom Dahl
Ari Zimran

* At the time the current analysis took place.

Table S2. GOS IRB locations.

Country<br>Site #<br>Name
**Albania**
1. Komiteti i Etikës
**Argentina**
2. Comité Independiente de Etica en investigación clínica "Dr. Carlos A. Barclay<br>3. Comisión Conjunta de Investigación en Salud (CCIS)<br>Comité de Bioética CIMeL
**Austria**
4. Ethikkommission der Medizinischen Universität Wien
**Brazil**
5. Comitê de Ética em Pesquisa do Hospital de Clínicas de Porto Alegre
**Canada**
6. Mount Sinai Hospital Research Ethics Board
**France**
7. Comité Consultatif sur le Traitement de l'Information en matière de Recherche dans le domain Santé<br>Commission Nationale de l'Informatique et des Libertés
**Germany**
8. Ethik-Kommission der Landesärztekammer Rheinland-Pfalz<br>9. Ethikkommission an der medizinischen Fakultät der Heinrich-Heine-Universität
**Israel**
10. Rambam Medical Center Institutional Helsinki Committee<br>11. Shaare Zedek Medical Center EC<br>12. Rabin Medical Center Local EC

Italy

13. Comitato Etico Regionale Unico (CERU)
    Comitato Etico Indipendente, Centro di Riferimento Oncologico – IRCCS

Korea, Republic of

14. Chungnam National University Hospital IRB
15. Asan Medical Center Institutional Review Board

Paraguay

16. Comité de Ética de la Investigación Científica, Docencia e Investigación Hospital Central del IPS

Poland

17. Komisja Bioetyczna przy Instytucie Pomnik Centrum Zdrowia Dziecka w Warszawie

Russian Federation

18. Ethics Committee at the FGBU NMITs for Haematology of the MoH of Russia

Spain

19. CEIC de Aragon (CEICA)
    Instituto Aragones de ciencias de la salud

Taiwan, Province of China

20. China Medical University and Hospital Research Ethics Committee
21. The Institutional Review Board of China Medical University Hospital

United Kingdom

22. Great Ormond Street Hospital for Children NHS Foundation Trust R&D
    Health Research Authority
    East of England - Cambridge South
23. Central Manchester University Hospitals NHS Foundation Trust R&D
    East of England - Cambridge South
24. Cambridge University Hospitals NHS Foundation Trust R&D
    East of England - Cambridge South
25. Royal Free London NHS Foundation Trust R&D
    East of England – Cambridge South

United States

26. Kaiser Permanente Southern California Institutional Review Board.
